# Long-Term Prediction of Emergency Department Revenue and Visitor Volume Using Autoregressive Integrated Moving Average Model

**DOI:** 10.1155/2011/395690

**Published:** 2011-12-04

**Authors:** Chieh-Fan Chen, Wen-Hsien Ho, Huei-Yin Chou, Shu-Mei Yang, I-Te Chen, Hon-Yi Shi

**Affiliations:** ^1^Emergency Department, Kaohsiung Municipal United Hospital, Kaohsiung 80457, Taiwan; ^2^Department of Health Business Administration, Meiho University, Pingtung 91202, Taiwan; ^3^Department of Healthcare Administration and Medical Informatics, Kaohsiung Medical University, Kaohsiung 80708, Taiwan; ^4^Center for General Education, Kaohsiung Medical University, Kaohsiung 80708, Taiwan

## Abstract

This study analyzed meteorological, clinical and economic factors in terms of their effects on monthly ED revenue and visitor volume. Monthly data from January 1, 2005 to September 30, 2009 were analyzed. Spearman correlation and cross-correlation analyses were performed to identify the correlation between each independent variable, ED revenue, and visitor volume. Autoregressive integrated moving average (ARIMA) model was used to quantify the relationship between each independent variable, ED revenue, and visitor volume. The accuracies were evaluated by comparing model forecasts to actual values with mean absolute percentage of error. Sensitivity of prediction errors to model training time was also evaluated. The ARIMA models indicated that mean maximum temperature, relative humidity, rainfall, non-trauma, and trauma visits may correlate positively with ED revenue, but mean minimum temperature may correlate negatively with ED revenue. Moreover, mean minimum temperature and stock market index fluctuation may correlate positively with trauma visitor volume. Mean maximum temperature, relative humidity and stock market index fluctuation may correlate positively with non-trauma visitor volume. Mean maximum temperature and relative humidity may correlate positively with pediatric visitor volume, but mean minimum temperature may correlate negatively with pediatric visitor volume. The model also performed well in forecasting revenue and visitor volume.

## 1. Introduction

Overcrowding in emergency departments (EDs) reflects dysfunction in healthcare systems [1]. Contributing factors including mismatch between ED capacity and various input, throughput, and output factors as well as insufficient capacity [2]. During the 12-year period from 1995 to 2006, annual ED visits in Taiwan increased 40%, from 4,664,209 to 6,569,247 per year [3]. Therefore, higher than expected admissions of critical patients to inpatient units is an important hospital administration issue [4].

Accurately predicting patient admissions can facilitate the timely planning of staff deployment and resource allocation in a department and in the entire hospital [5]. Although most medical organizations attempt to predict hourly or daily patient admissions, no studies have reported monthly forecasts of ED revenue, and very few have reported monthly forecasts of ED visitor volume. Moreover, no studies have simultaneously evaluated the possible associations of meteorological, clinical, and economic factors with ED revenue and visitor volume. Additionally, although the annual budget is a major consideration in hospital management, excellent care quality has the highest priority. Since ED medical services can easily incur large budget deficits, accurate revenue prediction provides the data needed to adjust budgets accordingly so that health care providers can allocate sufficient resources in advance. This study therefore analyzed the effects of meteorological, clinical, and economic factors on monthly ED revenue and visitor volume.

## 2. Materials and Methods

### 2.1. Study Design and Setting

This retrospective study was performed at the ED of a regional teaching hospital with 226 acute-care beds in Taiwan (22°N 120°E). Monthly data were analyzed for the period January 1, 2005, through September 30, 2009. A four-year (2005–2008) data set was used to construct the forecasting model, while the data for the first 9 months of the 5th year (2009) was used to test the forecasting capability of the model.

The ED visits were classified as trauma, nontrauma, or pediatric. All pediatric trauma patients and gynecology-obstetric trauma patients were initially treated by the trauma division. Nontrauma patients who were younger and older than 18 years were further classified as pediatric and nonpediatric nontrauma patients, respectively.

Because Kaohsiung city is located in a monsoon region and has a subtropical climate, dramatic monthly changes in temperature, humidity, and rainfall are common. Average monthly temperature ranges from 18.6 to 28.7 degrees Celsius, and average monthly humidity ranges from 60% to 81%. According to 1971–2000 data, average annual rainfall is approximately 1,785 mm. Although the hospital information system (HIS) had been implemented at the study facility since 2002, data collection was limited to the period from January, 2005 to September, 2009, due to the 2003 outbreak of severe acute respiratory syndrome.

### 2.2. Data Collection and Analysis

Potential predictors were selected according to the literature, local observation, and availability of data. Revenue data were provided by the hospital accounting department. Meteorological, clinical, and economic data were obtained from the Taiwan Central Weather Bureau (TCWB), Hospital Information System (HIS), and Taiwan Stock Exchange, respectively [6, 7].

According to the Financial Supervisory Commission, private investor transactions comprised more than 80% of all stock market investments during 2005–2009 [8]. The total number of investor accounts reached 15,143,707 in September, 2009, which represents 82.42% of the Taiwan adult population (18,374,613) [8]. Therefore fluctuation in the stock market index affected the economic status of most adults. The final model included the following factors: mean maximum temperature, mean minimum temperature, relative humidity, accumulated rainfall, and fluctuation in the stock market index. These databases are registered to the Taiwan Data Protection Authority for medical and research purposes. Given its design, aggregating data analysis with no individual identifiers, this study was exempted from the individual informed consent requirement.

 Spearman correlation analysis was used to test independent variables for correlations with case number. Moreover, given the potential lagged effect of the meteorological, clinical, and economic factors on ED revenue and visitor volume, cross-correlation analysis was also performed with relevant time lag values. Methods developed by Box and Jenkins [9] were used to build an autoregressive integrated moving average (ARIMA) time series model, which is designed to examine sequentially lagged relationships for relationships that may not be apparent in data collected periodically. The general form of the ARIMA model is

(1) $$D_{1}z_{t}=F_{1}z_{t-1}+\cdots +F_{p}z_{t-p}+a_{t}-q_{1}a_{t-1}-\cdots -q_{q}a_{t-q},$$

where *D*_1_ *z*_*t*_ = differenced series, that is,  *z*_*t*_ − *z*_*t*−1_, *zt* = set of possible observations of the time-sequenced random variable, *a*_*t*_ = random shock term at time *t*, *F*_1_ ⋯ *F*_*P*_ = autoregressive parameters of order *p*, *q*_1_ … *q*_*p*_ = moving average parameters of order *q*.

The series was subjected to Box-Cox transformation [10]. The transformed series was then differentiated at the nonseasonal level and mean corrected to induce stationarity. Sample autocorrelation and partial autocorrelation functions were used to identify the ARIMA model of the appropriate order. Model parameters were estimated by maximum likelihood method. Diagnostic tests, including residual analysis and the mean absolute percentage of error (MAPE), were performed to compare goodness-of-fit among ARIMA models. The final model obtained after several iterations of the identification, estimation, and checking processes met the conventional criteria for model adequacy.

To reflect changes in real dollar value, ED revenue data were adjusted by the consumer price index (CPI) for each year of 2005–2009 (95.16, 95.72, 97.44, 100.88, and 100.00, resp.). The ED revenues were then converted from Taiwan dollars to US dollars at an exchange rate of 30.5 : 1, which was the average exchange rate during 2005–2009. All tests were two-sided, and *P* values less than 0.05 were considered statistically significant. Statistical analysis was performed with SPSS software for Windows, version 15 (SPSS, Inc, Chicago, Ill, USA).

## 3. Results

**Figure 1:**
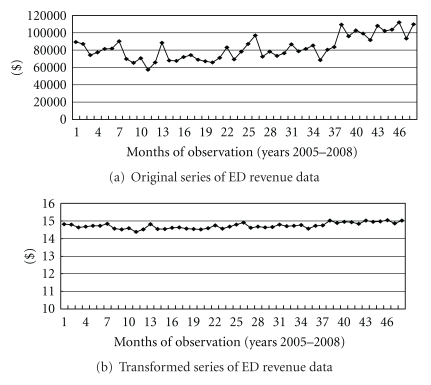
Original series and transformed series of emergency department (ED) revenue data for 2005 to 2008.

**Figure 2:**
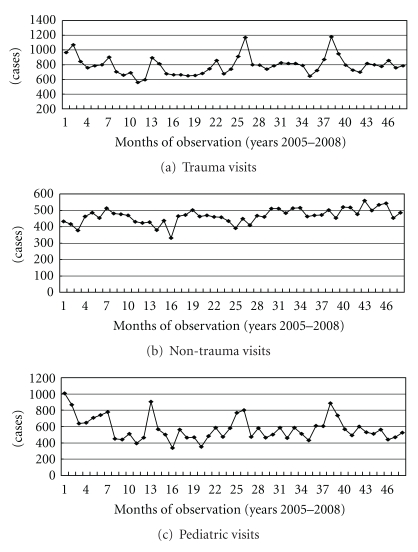
Original series data for trauma, non-trauma, and pediatric visits from 2005 to 2008.

After ED revenue adjustment and natural log processing, the series revealed good stability (Figure 1). Generally, the original series of trauma, nontrauma, and pediatric visits were stable (Figure 2). Although several peaks were noted in the three divisions and meteorological aspects, spectral analysis revealed no seasonal trends. The annual numbers of ED visitors from 2005 to 2008 were 22988, 20956, 22736, and 23416, respectively.

Table 1 summarizes the variables for meteorological, clinical, and economic conditions in Taiwan during the study period. Mean maximum temperature ranged from 27.85°C to 35.74°C, and mean minimum temperature ranged from 8.90°C to 25.22°C. Monthly relative humidity was 62.96%–91.18%, and monthly rainfall was 18.73 mm to 568.93 mm with maximum rainfalls observed in June and September. Additionally, the largest fluctuation in the stock index occurred in 2008.

The Spearman correlation analyses suggested that mean maximum temperature, relative humidity, accumulated rainfall, and stock index fluctuation were all positively correlated while mean minimum temperature was negatively correlated with monthly ED revenue, number of nontrauma visits, and number of pediatric visits, with lag time ranging from zero to two months (Table 2). Mean minimum temperature, accumulated rainfall, and stock index fluctuation were all positively correlated whereas mean maximum temperature and relative humidity correlated negatively with number of monthly trauma visits, with the lag time ranging from zero to two months. 

Table 3 shows the parameter estimates for the optimum ARIMA mode (1, 0, 0) for the series of monthly ED revenue. The autocorrelation and partial autocorrelation functions of the residuals showed a good data fit (data not shown). The residual plots showed small variations around the zero mean. In no case did the magnitude of these residuals exceed double the standard deviation. As a set, autocorrelations for residuals did not significantly differ from zero, and variance was consistent, which confirmed the adequacy of the model (Ljung-Box statistic = 22.04; *P* = 0.483). The analysis showed that mean maximum temperature, relative humidity, accumulated rainfall, nontrauma visits, and trauma visits were significantly and positively related to ED revenue, but mean minimum temperature was significantly and negatively related to ED revenue (*P* < 0.05). 

Table 4 shows the parameter estimates for the optimal ARIMA modes for the series of trauma visits, nontrauma visits, and pediatric visits. The autocorrelation and partial autocorrelation functions of the residuals also showed good data fit (data not shown). Mean minimum temperature and stock index fluctuation were significantly and positively associated with number of trauma visits (*P* < 0.05). Moreover, mean maximum temperature, relative humidity, and fluctuation in stock index were significantly and positively associated with number of nontrauma visits (*P* < 0.05). Additionally, mean maximum temperature and relative humidity were significantly and positively associated with number of pediatric visits, but mean minimum temperature was significantly and negatively associated with number of pediatric visits (*P* < 0.05). 

Table 5 shows that the performance of the ARIMA during validation phase was good to excellent. The validation phase data in Table 5 confirm the good forecasting capability of the ARIMA model. The model obtained a MAPE of 22.61% for ED revenue, 12.39% for trauma visits, 19.59% for nontrauma visits, and 29.08% for pediatric visits. 

## 4. Discussion

This study is the first to apply the ARIMA model for simultaneous time series analysis of three aspects of monthly ED revenue and patient visit. This study demonstrates that meteorological, clinical, and economic conditions affect ED revenue and patient visits. The model can be used for planning ED staff deployments and for resource allocation. It can also forecast and resolve inadequate capacity in EDs. 

Previous studies of ED revenue only compared difference between weekdays and weekends [11]. Understanding the deficit from ED operation, the hospital manager can arrange appropriate budget in advance. This study found that ED revenue correlated positively with mean maximum temperature, relative humidity, accumulated rainfall, number of trauma, and nontrauma visits, but negatively mean minimum temperature. Although rainfall was significantly associated with revenue, it was unassociated with volume of trauma, nontrauma, and pediatric patients. A possible explanation is that the severity of diseases treated at EDs increases during rainy season [12]. The number of health insurance claims during the study period was not significantly related to patient volume. 

Interestingly, stock index fluctuation correlated positively with overall patient volume (trauma and nontrauma), which correlated positively with monthly ED revenue. The lack of correlation between stock index fluctuation and ED revenue may be due to the reduced effect of ED revenue when simultaneously considering multiple factors in the forecasting model. 

Although many recent studies have evaluated the effect of climate change on human health, few studies have considered multiple factors associated with human health [12–14]. The effects of meteorological conditions on specific diseases have already been demonstrated [15]. Therefore, this study evaluated the effect of meteorological conditions on all ED patients treated in one facility. In contrast with previous reports, mean minimum temperature was associated with number of trauma patients, and mean maximum temperature was associated with number of nontrauma patients [15, 16]. The effects of weather changes on residents in different regions may explain the difference [17]. Residents of tropical climates who are accustomed to warm temperatures may have low tolerance for cold temperatures. Thus, cold weather may cause people in tropical climates to hurry and drive less carefully. Driving at high speeds is another major cause of traffic accidents in Taiwan, especially those involving motorcycles. Hence, trauma-causing accidents may increase during cold weather in Taiwan. Wearing thin clothes on days with high temperatures may also contribute to the risk of motorcycle injuries [18].

Although low temperature was associated with severe medical and pediatric disease, maximum temperatures also correlated with the incidence of Dengue fever, pediatric fever, and gastroenteritis [19, 20]. Other studies have reported that relative humidity correlates with pulmonary disease outbreaks, acute upper respiratory infection, and respiratory syncytial virus (RSV) infection [14, 21, 22]. Therefore, mean maximum temperature and relative humidity correlate with patient visits in both nontrauma and pediatric divisions. Relative humidity may also explain regional differences [23].

Time series analysis with ARIMA model is an accurate method of long-term forecasting [24–26]. This study demonstrated good-to-excellent accuracy in forecasting monthly ED revenue, nontrauma visits, trauma visits, and pediatric visits. Since most clinics and hospital out-patient departments are closed during the traditional Chinese new year period, ED visits tend to increase in most medical institutions. Notably, Chinese new year fell in February during 2005~2008, but it fell in January in 2009. When forecasting accuracy was considered only for February to September, 2009, a dramatic improvement was observed. In forecasts of trauma visits, the best MAPE was 10.16%, and the worst MAPE was 13.11%. In forecasts of nontrauma visits, the best MAPE was 13.71%, and the worst MAPE was 21.46%. In forecasts of pediatric visits, the best MAPE was 5.73% (for the February, 2009 forecast), and the worst MAPE was 54.24% (for the September, 2009 forecast). Except for September, 2009, all forecasts for pediatric visits during February~August, 2009 had MAPEs of 5.73%~21.18%. The September deviation resulted mainly from an H1N1 influenza outbreak in teenagers. 

The main objective of business forecasting is appropriately adjusting staffing to business activity, which in this case was ED activity. The forecasts indicated that one nurse, one emergency physician, and computer equipment should have been added during December, 2009, to February, 2010. Average waiting time decreased from 21 minutes to 13 minutes, and average length of stay in the triage categories of life-threatening and emergent decreased from 124 minutes to 117 minutes. The number of patients referred to other hospitals decreased from 23 in December, 2009, to 9 in February, 2010; during the same period, the number of patients treated monthly increased from 2,483 to 3,207, and the percentages of monthly admissions increased from 37.13% to 48.09%. However, in the one study in the current literature that has performed a numerical analysis to optimize staffing, an 18.5% decrease in patients who “left without treatment” was used as a surrogate marker [27].

One limitation of this study is that the patients were treated in a regional teaching hospital in Taiwan, where almost all citizens have national health insurance with unrestricted access to emergency care. This should be considered when generalizing the findings of the study to other hospitals. Second, this study did not explore socioeconomic indicators other than stock index fluctuation. Third, patient and staff satisfaction was not included in the assessment of the effect of adjustment results.

## 5. Conclusions

Emergency departments must continue operating even when insufficient capacity causes overcrowding. Meteorological, clinical, and economic factors are associated with ED revenue and visitor volume. The good long-term forecasting capability of the model proposed in this study can help EDs to optimize departmental resources and manpower. Emergency services can also be enhanced by matching-associated input and throughput factors.

## Figures and Tables

**Table 1:** Summary of monthly related variables from January, 2005-December, 2008.

Variable	Mean	Std. Dev.	Min.	Max.	F*
Mean maximum temperature	31.04	10.21	27.85	35.74	9.73
Mean minimum temperature	18.44	4.53	8.90	25.22	16.52
Relative humidity	78.09	12.14	62.96	91.18	41.60
Accumulated rainfall	353.23	108.06	18.73	568.93	10.69
Stock index fluctuation	678.35	122.90	280.33	1020.44	30.32

*A *P* value <0.05 was considered statistically significant.

**Table 2:** Spearman correlation between ED revenue, trauma, nontrauma, and pediatric visits and related variables (January, 2005–December, 2008).

Variable	ED revenue		Trauma visit		Nontrauma visit		Pediatric visit	
	Coefficient 95% CI	Lag values	Coefficient 95% CI	Lag values	Coefficient 95% CI	Lag values	Coefficient 95% CI	Lag values
Mean maximum temperature	0.38 (0.33, 0.43)**	1 month	−0.22 (−0.37, −0.07)*	0 month	0.45 (0.37, 0.53)**	1 month	0.31 (0.23,0.38)**	1 month
Mean minimum temperature	−0.45 (−0.51, −0.39)**	1 month	0.41 (0.31, 0.52)**	1 month	−0.33 (−0.45, −0.22)*	1 month	−0.38 (−0.45, −0.32)**	1 month
Relative humidity	0.48 (0.40, 0.56)**	0 month	−0.52 (−0.70, −0.35)*	1 month	0.23 (0.06, 0.39)*	0 month	0.34 (0.20, 0.57)*	1 month
Accumulated rainfall	0.47 (0.38, 0.56)**	2 month	0.28 (0.11, 0.46)*	1 month	0.12 (0.01, 0.22)*	1 month	0.23 (0.10, 0.36)*	2 month
Stock index fluctuation	0.18 (−0.03, 0.39)*	1 month	0.30 (0.20, 0.39)**	2 month	0.27 (0.20, 0.34)**	1 month	0.44 (0.30, 0.59)*	1 month

*P < 0.05, **P < 0.01, statistically significant.

**Table 3:** Parameters from autoregressive integrated moving average (ARIMA) model (1, 0, 0) for ED revenue (January, 2005–December, 2008).

Parameters	Coefficient	*T*	*P* value
Mean maximum temperature	0.1176	2.77	0.009
Mean minimum temperature	−0.0736	−5.29	<0.001
Relative humidity	0.0672	4.33	<0.001
Accumulated rainfall	0.0008	4.18	<0.001
Nontrauma visits	0.0040	3.38	0.002
Trauma visits	0.0098	5.38	<0.001
Pediatric visits	−0.0004	−0.78	0.442
Stock index fluctuation	−0.0002	−0.74	0.463

**Table 4:** Parameters from autoregressive integrated moving average (ARIMA) model for trauma, nontrauma, and pediatric visits (January, 2005–December, 2008).

Parameters	Coefficient	*T*	*P* value
ARIMA model (1,0,2) for forecasting trauma visits			
Mean maximum temperature	−6.2110	−1.545	0.131
Mean minimum temperature	6.8860	4.383	<0.001
Relative humidity	−0.5100	−0.360	0.721
Accumulated rainfall	0.0210	1.479	0.147
Stock index fluctuation	0.0990	5.026	<0.001

ARIMA model (1,0,2) for forecasting nontraumatic visits			
Mean maximum temperature	0.1380	5.211	<0.001
Mean minimum temperature	−0.0120	−0.835	0.409
Relative humidity	0.0280	2.518	0.016
Accumulated rainfall	0.0001	0.281	0.780
Stock index fluctuation	0.0010	3.351	0.002

ARIMA model (0, 2, 1) for forecasting pediatric visits			
Mean maximum temperature	0.1320	3.449	<0.001
Mean minimum temperature	−0.0065	−4.444	<0.001
Relative humidity	0.0040	2.552	0.015
Accumulated rainfall	0.0001	0.921	0.363
Stock index fluctuation	0.0001	1.606	0.116

**Table 5:** Prediction results of autoregressive integrated moving average (ARIMA) models in 2009.

Date	ED revenue		Traumatic visit		Nontraumatic visit		Pediatric visit	
	True Value	Forecasted value	True value	Forecasted value	True value	Forecasted value	True value	Forecasted value
Jan-09	4,767,559	3,676,314	584	472	1,415	826	1,204	551
Feb-09	3,885,639	4,547,216	465	498	1,162	1,177	630	699
Mar-09	3,419,070	3,529,467	597	535	1,090	857	595	719
Apr-09	3,897,391	3,313,426	582	507	945	910	546	740
May-09	3,804,037	2,158,604	572	502	962	862	613	712
Jun-09	3,336,949	5,126,700	570	539	852	946	491	657
Jul-09	3,642,391	5,576,091	599	575	903	845	476	560
Aug-09	4,703,707	5,731,772	692	590	1,179	917	802	548
Sep-09	5,058,538	5,787,666	675	625	1,366	1,047	1,324	787

MAPE	22.61% (14.38%~29.73%)		12.39% (10.16%~19.12%)		19.59% (13.71%~41.61%)		29.08% (5.73%~54.24%)	

MAPE: mean absolute percentage of error.
